# Little Fish Are Less Likely to Take the Bait

**DOI:** 10.1371/journal.pmed.0020221

**Published:** 2005-07-26

**Authors:** Harvey Marcovitch

**Affiliations:** **1**BMJ Publishing GroupLondonUnited Kingdom

One solution for fair-minded doctors not mentioned by Smith [1] might be to keep away from major high-impact journals and subscribe instead to those with a lower profile but that serve their specialty. I analysed all original papers published in the last 12 issues of *Archives of Disease in Childhood*. Of 198 such papers, there were seven (3.5%) manufacturer-funded studies dealing with drugs, vaccines, or infant foods. Another ten papers (5%) dealt with drugs or vaccines, including three reports of adverse events, but were not funded by industry. The funding of one was obscure. This pristine record was somewhat spoiled by a sponsored supplement, but clearly labelled as such, about a particular medication. It provoked an angry correspondence on the subscribers' message board of one of the co-publishers. It seems that at least paediatrics, a far-away specialty of which Smith may know little, treads a careful path.
